# A novel predictive tool for pulmonary aspergillosis in critically ill patients with viral pneumonia: a multi-center, ambispective cohort study

**DOI:** 10.3389/fcimb.2026.1956822

**Published:** 2026-10-27

**Authors:** Jing Zhang, Shengnan Xu, Leilei Guo, Bo Qiao, Yuanyuan Li, Yansheng Zhou, Xiufeng Hu, Yanyan Guo, Bin Hu, Sangang Zheng, Chao Chen, Yajun Li, Junping Liu, Jiangfeng Zhang, Peng Li

**Affiliations:** 1 Respiratory Intensive Care Unit, Henan Provincial People’s Hospital, People’s Hospital of Zhengzhou University, Zhengzhou, China; 2 Infection Prevention and Control Department, Zhengzhou Central Hospital Affiliated to Zhengzhou University, Zhengzhou, China; 3 Infection Prevention and Control Department, Henan Provincial Chest Hospital, Zhengzhou, China; 4 Infection Prevention and Control Department, The First Affiliated Hospital of Nanyang Medical College, Nanyang, China; 5 Infection Prevention and Control Department, The First Affiliated Hospital of Henan Medical University, Xinxiang, China; 6 Infection Prevention and Control Department, The First Affiliated Hospital of Henan University of Science and Technology, Luoyang, China; 7 Infection Prevention and Control Department, Huaihe Hospital of Henan University, Kaifeng, China; 8 Infection Prevention and Control Department, Zhumadian Central Hospital, Zhumadian, China; 9 Infection Prevention and Control Department, Jiyuan People’s Hospital, Jiyuan, China; 10 Central Intensive Care Unit, Henan Provincial People’s Hospital, People’s Hospital of Zhengzhou University, Zhengzhou, China; 11 Department of Infectious Disease, Henan Provincial People’s Hospital, People’s Hospital of Zhengzhou University, Zhengzhou, China; 12 Department of Clinical Microbiology, Henan Provincial People’s Hospital, People’s Hospital of Zhengzhou University, Zhengzhou, China; 13 Department of Infection Control, Henan Provincial People’s Hospital, People’s Hospital of Zhengzhou University, Zhengzhou, China

**Keywords:** competing risk, critically ill, prediction, pulmonary aspergillosis, viral pneumonia

## Abstract

**Background:**

Viral pneumonia-associated pulmonary aspergillosis (VAPA) is a lethal complication in critically ill patients. We aimed to develop a generalizable and methodologically robust prediction tool for early VAPA risk, overcoming key limitations of existing models.

**Methods:**

In a multi-center, ambispective cohort of 4,966 patients with influenza or COVID-19 admitted to ICU (Jan 2023–Jun 2025), we developed and validated two prediction models for VAPA using LASSO-selected variables: a standard Cox proportional hazards model and a Fine-Gray subdistribution hazards model accounting for non-VAPA death as a competing risk. For both models, restricted cubic splines were used to address nonlinear associations between continuous predictors and VAPA. Performance was assessed at 7 and 14 days post-ICU admission. Subgroup analysis was employed to examine the virus-specific models’ performance.

**Results:**

The incidence of VAPA was 8.8%. The Fine-Gray model demonstrated excellent discrimination and calibration in both derivation and validation cohorts. In contrast, the standard Cox model substantially overestimated VAPA risk, particularly among high-risk patients (by 20-30%). Key predictors included haematological malignancy, halo sign, use of priority antibiotic combinations, SOFA score, neutrophil-to-lymphocyte ratio (NLR), and pre-ICU corticosteroid dose; the latter three exhibited J-shaped associations with VAPA risk. A significant interaction was observed between viral subtype and pre-ICU corticosteroid dose (*P* = 0.006), with a stronger association in COVID-19 patients (sHR=1.17, 95% CI: 1.09-1.26). Overall model performance remained consistent across viral subtypes. An interactive web-based tool was then deployed, which generates both individualized VAPA risk estimates and corresponding clinical management recommendations.

**Conclusions:**

In critically ill patients with viral pneumonia, a competing-risk model accounting for non-VAPA death provides more accurate estimates of VAPA risk than traditional approaches. The model demonstrates robust generalizability across influenza and COVID-19 pneumonia and offers a practical tool for early, personalized VAPA prediction.

## Introduction

Viral pneumonia, particularly from influenza and SARS-CoV-2, is a leading cause of critical illness and mortality worldwide. The clinical course of these patients is often complicated by secondary infections, among which viral pneumonia-associated pulmonary aspergillosis (VAPA)—encompassing influenza-associated (IAPA) and COVID-19-associated (CAPA) forms—has emerged as a particularly lethal complication (Feys et al., 2024). With reported incidences of 10%–20% in critically ill patients, VAPA carries an attributable mortality of nearly 50% (Feys et al., 2024). However, the accurate and timely diagnosis of VAPA remains challenging due to non-specific clinical features, ambiguous radiological signs, and the difficulties in obtaining definitive mycological evidence (Egger et al., 2022; Hatzl et al., 2025). This diagnostic dilemma often leads to delays in initiating appropriate antifungal therapy, highlighting the urgent need for reliable and effective risk stratification tools.

Nevertheless, critical gaps persist in current VAPA risk prediction. Most existing models are primarily restricted to single viral etiologies (e.g., CAPA-specific) and thus lack generalizability across the spectrum of viral pathogens (Gioia et al., 2024; Lu et al., 2024). More fundamentally, they methodologically treat non-VAPA death—whether resulting from the primary viral infection or underlying comorbidities—as a censoring event rather than a competing risk (Liu et al., 2025). This conventional approach overlooks the fact that death precludes the occurrence of VAPA, leading to a potentially biased estimation of its absolute risk. Such bias is particularly pronounced in critically ill patients and may prompt overtreatment and inefficient allocation of critical care resources (Van Geloven et al., 2022). To bridge these gaps, we employed a multi-center, ambispective cohort to evaluate a competing risk model that takes into account the non-VAPA death in predicting VAPA, and to develop and validate a novel, dynamic predictive tool for guiding targeted prevention and early intervention in clinical practice.

## Methods

### Study design and data collection

This ambispective cohort study was conducted across 48 ICUs from 9 public tertiary hospitals in Central China. We enrolled adult patients (aged >18 years) with confirmed diagnosis of viral pneumonia (influenza or COVID-19) who were admitted to the ICU for ≥ 48 hours between January 2023 and June 2025. Patients were excluded if they had a pre-existing history of pulmonary aspergillosis or if documentation of their infection status during the ICU stay was unavailable. Data for the 54 candidate predictor variables were collected at or within 24 hours of ICU admission from the Hospital and Laboratory Information Systems (HIS/LIS) of the participating centers. Follow-up started at ICU admission and ended at the earliest occurrence of one of the following: diagnosis of VAPA, death from a non-VAPA cause, or discharge from the ICU without a VAPA diagnosis. The overall cohort was temporally divided into a derivation cohort (January 2023 to June 2024) for model training and internal validation, and a prospective validation cohort (July 2024 to June 2025) for external temporal validation (**Figure 1**).

**Figure 1:**
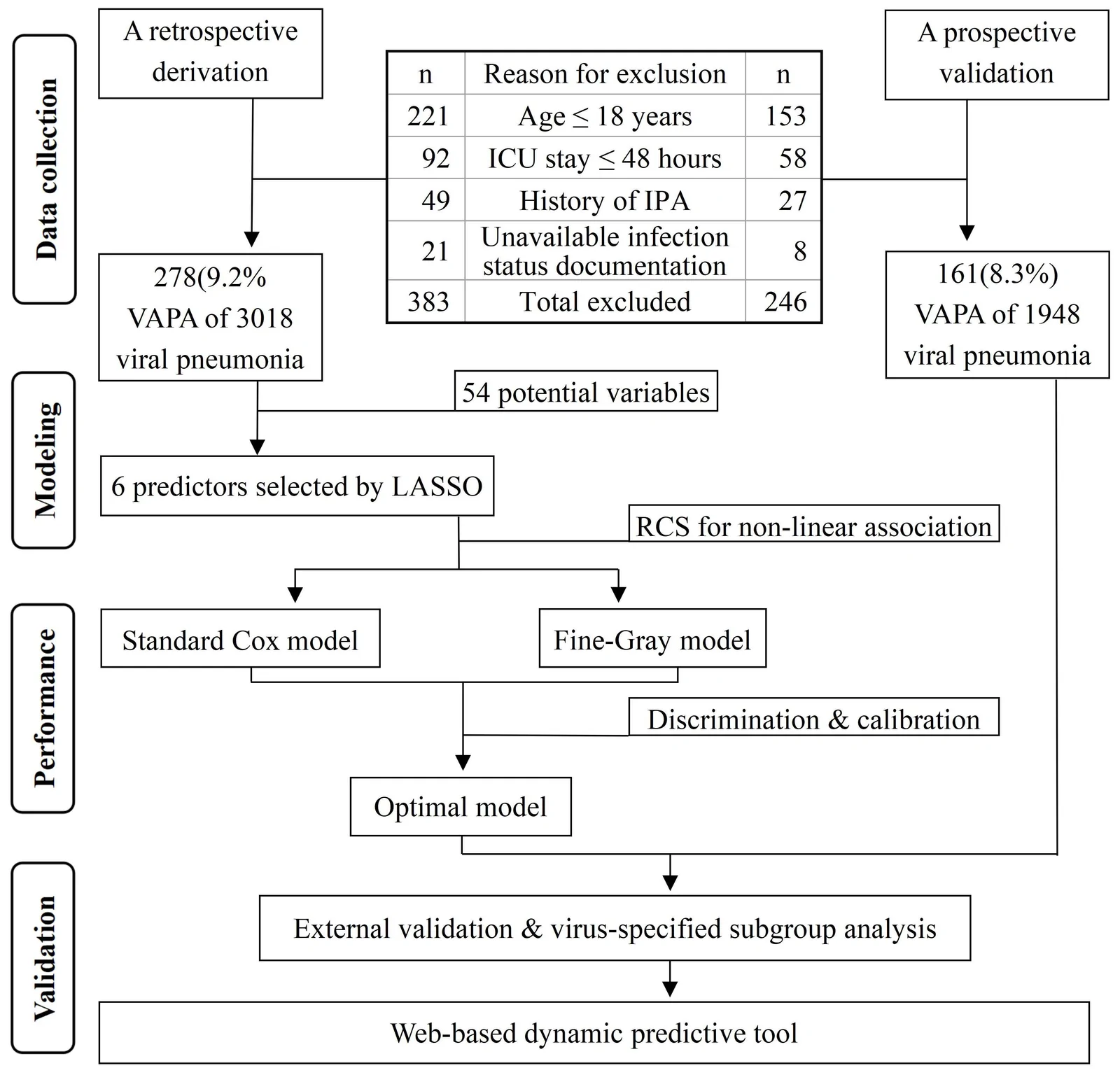
Flow diagram of study cohort and prediction modeling.

The study protocol was approved by the ethics committees of all participating hospitals (lead registration number: HNSRY2023-37). And due to the non-interventional, observational nature of the research and the use of fully anonymized patient data, the requirement for individual informed consent was waived.

### Outcome and exposure definitions

The primary outcome was the occurrence of proven or probable VAPA during the ICU stay. Cases were defined using the expert criteria by Verweij et al. for IAPA and the 2020 European Confederation of Medical Mycology/International Society for Human and Animal Mycology (ECMM/ISHAM) consensus criteria for CAPA, respectively (Verweij et al., 2020; Koehler et al., 2021).

Some exposure variables were defined as follows: (1) Vaccination within the prior year: For patients with influenza, this referred to administration of the influenza vaccine; for patients with COVID-19, it referred to administration of a COVID-19 vaccine, in each case within one year before ICU admission. (2) Bacterial co-infection: Defined as the detection of a pathogenic bacterium from respiratory tract specimens obtained during ICU admission screening. (3) Viral co-infection: Defined as the detection of two or more distinct respiratory viruses (e.g., influenza, SARS-CoV-2, rhinovirus, adenovirus, or respiratory syncytial virus) via multiplex PCR from nasopharyngeal or lower respiratory tract specimens obtained during ICU admission screening, with at least one of the viruses being influenza or SARS-CoV-2. For virus-specific analyses, patients with influenza/SARS-CoV-2 co-infection were counted in both virus groups; similarly, co-infected patients who developed VAPA were counted in both IAPA and CAPA subtypes. (4) Priority antibiotic combinations: Defined as the concurrent use of two or more agents from the priority list issued by the National Institute of Hospital Administration of China, which included carbapenems, glycopeptides (teicoplanin and vancomycin), linezolid, tigecycline, polymyxin, and β-lactam/β-lactamase inhibitor combinations — representing broad-spectrum antibiotics, MRSA-targeting agents, and antibiotics for resistant Gram-negative organisms, respectively. (5) Cumulative corticosteroid dose before ICU admission: The total dose of corticosteroids (oral, injectable, or inhaled) administered for the viral pneumonia episode prior to ICU admission, and doses of different corticosteroids were converted to dexamethasone equivalents.

### Statistical analysis

The sample size was determined by all available data from the defined cohort period. Missing data were handled using the multiple imputation method with chained equations(MICE), as detailed in **Table 1**.

**Table 1:** Baseline characteristics of ICU patients with viral pneumonia.

Characteristics	Derivation cohort			Validation cohort		
	No VAPA (*N* = 2740)	VAPA (N = 278)	*P* value after MICE	No VAPA (*N* = 1787)	VAPA (*N* = 161)	*P* value after MICE
Host factors						
Age (years), mean ± SD	59.6 ± 5.7	63.4 ± 5.6	<0.001	59.7 ± 5.7	62.4 ± 5.2	<0.001
Sex (male), *n* (%)	1678 (61.2)	166 (59.7)	0.651	1098 (61.4)	96 (59.6)	0.673
BMI, mean ± SD	21.5 ± 4.1	21.3 ± 3.8	0.553	22.1 ± 4.1	21.8 ± 3.9	0.267
Smoking	533 (19.5)	78 (28.1)	0.001	348 (19.5)	35 (21.7)	0.470
Vaccination within prior year	156 (5.7)	21 (7.6)	<0.001	103 (5.8)	11 (6.8)	0.597
APACHE II, median (IQR)	18 [14–23]	19 [12-25]	0.102	18 [13-22]	18 [11-24]	0.578
SOFA, median (IQR)	7 [6-7]	9 [8-11]	<0.001	6 [6-7]	9 [7-11]	<0.001
aCCI, median (IQR)	5 [4-7]	6 [4-8]	0.050	5 [4-7]	6 [4-8]	0.060
Solid malignancy, n (%)	338 (12.3)	49 (17.6)	0.014	178 (10.0)	25 (15.5)	0.031
Haematological malignancy, n (%)	90 (3.3)	71 (25.5)	<0.001	66 (3.7)	34 (21.1)	<0.001
Solid organ transplant recipient, n (%)	110 (4.0)	14 (5.0)	0.426	70 (3.9)	8 (5.0)	0.526
Chronic liver disease, n (%)	117 (4.3)	16 (5.8)	0.281	89 (5.0)	5 (3.1)	0.342
Chronic kidney disease, n (%)	250 (9.1)	54 (19.4)	<0.001	136 (7.6)	40 (24.8)	<0.001
COPD, n (%)	415 (15.1)	79 (28.4)	<0.001	287 (16.1)	48 (29.8)	<0.001
Chronic interstitial lung, n (%)	451 (16.5)	40 (14.4)	0.395	288 (16.1)	19 (11.8)	0.175
Asthma, n (%)	182 (6.6)	28 (10.1)	0.046	113 (6.3)	16 (9.9)	0.095
Hypertension, n (%)	1326 (48.4)	119 (42.8)	0.078	885 (49.5)	72 (44.7)	0.250
Diabetes, n (%)	517 (18.9)	100 (36.0)	<0.001	367 (20.5)	61 (37.9)	<0.001
Cerebrovascular disease, n (%)	475 (17.3)	57 (20.5)	0.187	330 (18.5)	31 (19.3)	0.832
Cardiovascular disease, n (%)	1452 (53.0)	138 (49.6)	0.313	877 (49.1)	81 (50.3)	0.805
Bacterial co-infection, n (%)	934 (34.1)	175 (62.9)	<0.001	623 (34.9)	104 (64.6)	<0.001
Viral co-infection, n (%)	155 (5.7)	11 (4.0)	0.271	120 (6.7)	11 (6.8)	0.871
Clinical symptoms						
Fever > 38.5°C, n (%)	1524 (55.6)	169 (60.8)	0.100	1020 (57.1)	103 (64.0)	0.096
Chills, n (%)	215 (7.8)	26 (9.4)	0.355	145 (8.1)	8 (5.0)	0.171
Cough, n (%)	415 (15.1)	38 (13.7)	0.597	262 (14.7)	24 (14.9)	0.908
Sputum, n (%)	2209 (80.6)	237 (85.3)	0.065	1461 (81.8)	133 (82.6)	0.915
Hemoptysis, n (%)	141 (5.1)	20 (7.2)	0.160	99 (5.5)	21 (13.0)	0.001
Respiratory rate > 30/min, n (%)	2474 (90.3)	247 (88.8)	0.459	1609 (90.0)	144 (89.4)	0.784
Laboratory results						
Lymphocyte (10/L), median (IQR)	0.6 [0.4-0.8]	0.4 [0.3-0.6]	<0.001	0.6 [0.5-0.8]	0.4 [0.3-0.7]	<0.001
NLR, median (IQR)	6.6 [4.9-9.1]	11.1 [8.9-14.2]	<0.001	6.5 [4.8-8.9]	10.4 [8.0-12.6]	<0.001
CRP (mg/L), median (IQR)	110.0 [78.0-154.0]	107.5 [85.0-163.2]	0.219	109.0 [80.0-152.5]	118.0 [90.0-165.0]	0.018
PCT (ng/mL), median (IQR)	0.5 [0.3-0.8]	0.6 [0.4-1.2]	<0.001	0.5 [0.3-0.8]	0.7 [0.3-1.1]	<0.001
Imaging features						
Halo Sign, n (%)	189 (6.9)	55 (19.8)	<0.001	124 (6.9)	37 (23.0)	<0.001
Air-crescent Sign, n (%)	85 (3.1)	13 (4.7)	0.156	48 (2.7)	6 (3.7)	0.447
Cavitation, n (%)	152 (5.5)	24 (8.6)	0.043	90 (5.0)	10 (6.2)	0.459
Consolidation, n (%)	1423 (51.9)	164 (59.0)	0.027	947 (53.0)	90 (55.9)	0.510
Bronchial Wall Thickening, n (%)	586 (21.4)	55 (19.8)	0.590	367 (20.5)	41 (25.5)	0.156
Treatments						
Priority antibiotic combination, n (%)	483 (17.6)	144 (51.8)	<0.001	306 (17.1)	76 (47.2)	<0.001
Antifungal prophylaxis, n (%)	345 (12.6)	41 (14.7)	0.300	220 (12.3)	27 (16.8)	0.108
Pre-ICU corticosteroid cumulative dose (mg), median (IQR)	31 [18-46]	60.0 [52-84]	<0.001	31 [18-47]	64 [53-85]	<0.001
Pre-ICU corticosteroid days (d), median (IQR)	3 [1-4]	4 [2-6]	<0.001	3 [1-4]	3 [2-5]	0.001
Corticosteroid in ICU admission, n (%)	1312 (47.9)	221 (79.5)	<0.001	798 (44.7)	133 (82.6)	<0.001
Interleukin-6 inhibitors, n (%)	99 (3.6)	24 (8.6)	<0.001	67 (3.7)	12 (7.5)	0.034
Prone ventilation, n (%)	1660 (60.6)	149 (53.6)	0.025	1086 (60.8)	102 (63.4)	0.555
High-flow oxygen therapy, n (%)	1565 (57.1)	191 (68.7)	<0.001	1060 (59.3)	109 (67.7)	0.044
Invasive mechanical ventilation, n (%)	940 (34.3)	168 (60.4)	<0.001	614 (34.4)	96 (59.6)	<0.001
ECMO, n (%)	78 (2.8)	8 (2.9)	1.000	36 (2.0)	9 (5.6)	0.009
Dialysis, n (%)	795 (29.0)	106 (38.1)	0.002	540 (30.2)	49 (30.4)	1.000
Parenteral nutrition, n (%)	2064 (75.3)	206 (74.1)	0.662	1326 (74.2)	124 (77.0)	0.509
Environmental exposure						
HEPA filter in use, n (%)	593 (21.6)	51 (18.3)	0.219	420 (23.5)	28 (17.4)	0.079
Negative pressure, n (%)	89 (3.2)	26 (9.4)	<0.001	59 (3.3)	23 (14.3)	<0.001
Single room, n (%)	740 (27.0)	98 (35.3)	0.005	482 (27.0)	52 (32.3)	0.166
Admission source, n (%)			<0.001			0.012
Emergency	1014 (37.0)	75 (27.0)		678 (37.9)	43 (26.7)	
Community	429 (15.7)	32 (11.5)		278 (15.6)	23 (14.3)	
General wards	720 (26.3)	104 (37.4)		487 (27.3)	60 (37.3)	
Other hospitals	577 (21.1)	67 (24.1)		344 (19.3)	35 (21.7)	
Admission season, *n* (%)			0.791			0.933
Spring	563 (20.5)	51 (18.3)		342 (19.1)	28 (17.4)	
Summer	146 (5.3)	14 (5.0)		87 (4.9)	9 (5.6)	
Autumn	274 (10.0)	31 (11.2)		180 (10.1)	17 (10.6)	
Winter	1757 (64.1)	182 (65.5)		1178 (65.9)	107 (66.5)	

Two prediction models for VAPA were developed using predictors selected by least absolute shrinkage and selection operator (LASSO) regularization: a standard Cox proportional hazards model and a Fine-Gray subdistribution hazards model, the latter of which accounts for the competing risk of non-VAPA death. Both models incorporated restricted cubic splines (RCS) with four knots for continuous variables to capture potential nonlinear relationships. Model performance was internally validated in the derivation cohort and temporally validated in an external cohort at two clinically relevant time points—7 and 14 days post-ICU admission. Discrimination was assessed using time-dependent area under the receiver operating characteristic curve (AUC), and calibration was evaluated using calibration curves with bootstrap-based bias correction, comparing predicted against observed VAPA probabilities. Model comparisons were performed using DeLong’s test for AUC differences and Brier scores for overall predictive accuracy.

To examine the stability of the model across viral etiologies, we specifically tested for interaction effects between the virus subtypes and the predictors using a multiplicative interaction term approach within the Fine–Gray framework. We further performed a prespecified subgroup analysis in the validation cohort by separately assessing the virus-specific models’ performance among influenza and COVID-19 patients. To operationalize the final Fine-Gray model for use in clinical settings and future interventional research, we developed an interactive, real-time nomogram integrated with clinical decision support. This tool was deployed as a publicly accessible web application via shinyapps.io (Posit Software, PBC), enabling dynamic risk stratification and individualized management recommendations based on our model predictions.

This study followed the Transparent Reporting of a multivariable prediction model for Individual Prognosis Or Diagnosis (TRIPOD) statement (see **Supplementary Table 1** for the checklist). And the evaluation of the Fine-Gray model’s performance adhered to the guidance provided by the Strengthening Analytical Thinking for Observational Studies (STRATOS) initiative. All statistical analyses were conducted using R software (version 4.4.2 R Foundation for Statistical Computing), with a two-tailed significance level of α = 0.01.

## Results

### Characteristics of cohort

This study included 4,966 ICU patients with influenza (n=3,079) or COVID-19 (n=1,930) admitted between January 1, 2023, and June 30, 2025. Among them, 1,948 patients were prospectively enrolled from July 2024 to June 2025. Baseline characteristics within 24 hours of ICU admission are presented in **Table 1**. Overall, 439 patients (8.8%) developed VAPA, including 273 (8.9%) with IAPA and 185 (9.6%) with CAPA. Among the 43 patients with influenza/SARS-CoV-2 co-infection, 19 (44.2%) developed VAPA and were counted in both the IAPA and CAPA subgroups. Additionally, 177 patients (3.6%) died from non-VAPA causes.

### Predictors selection and models development

Following LASSO selection, 17 of the 54 candidate predictors were retained at the minimum mean-squared error (MSE), while 6 predictors (SOFA score, haematological malignancy, neutrophil-to-lymphocyte ratio [NLR], halo sign, use of priority antibiotic combinations, and cumulative corticosteroid dose before ICU admission) were selected under the more stringent criterion of minimum MSE plus one standard error (SE; **Figure 2**). For haematological malignancy, halo sign, and use of priority antibiotic combinations, the hazard ratios (HR) from the Cox model and subdistribution hazard ratios (sHR) from the Fine-Gray model were comparable (**Table 2**). Likelihood-ratio (LR) tests identified significant nonlinear relationships between VAPA risk and three continuous predictors: SOFA score (LR = 164.93, *P* < 0.01), NLR (LR = 22.00, *P* = 0.009), and cumulative corticosteroid dose before ICU admission (LR = 364.56, *P* < 0.01). When modeled with RCS, all three predictors consistently exhibited a J-shaped association with VAPA risk in both models (**Figure 3**).

**Figure 2:**
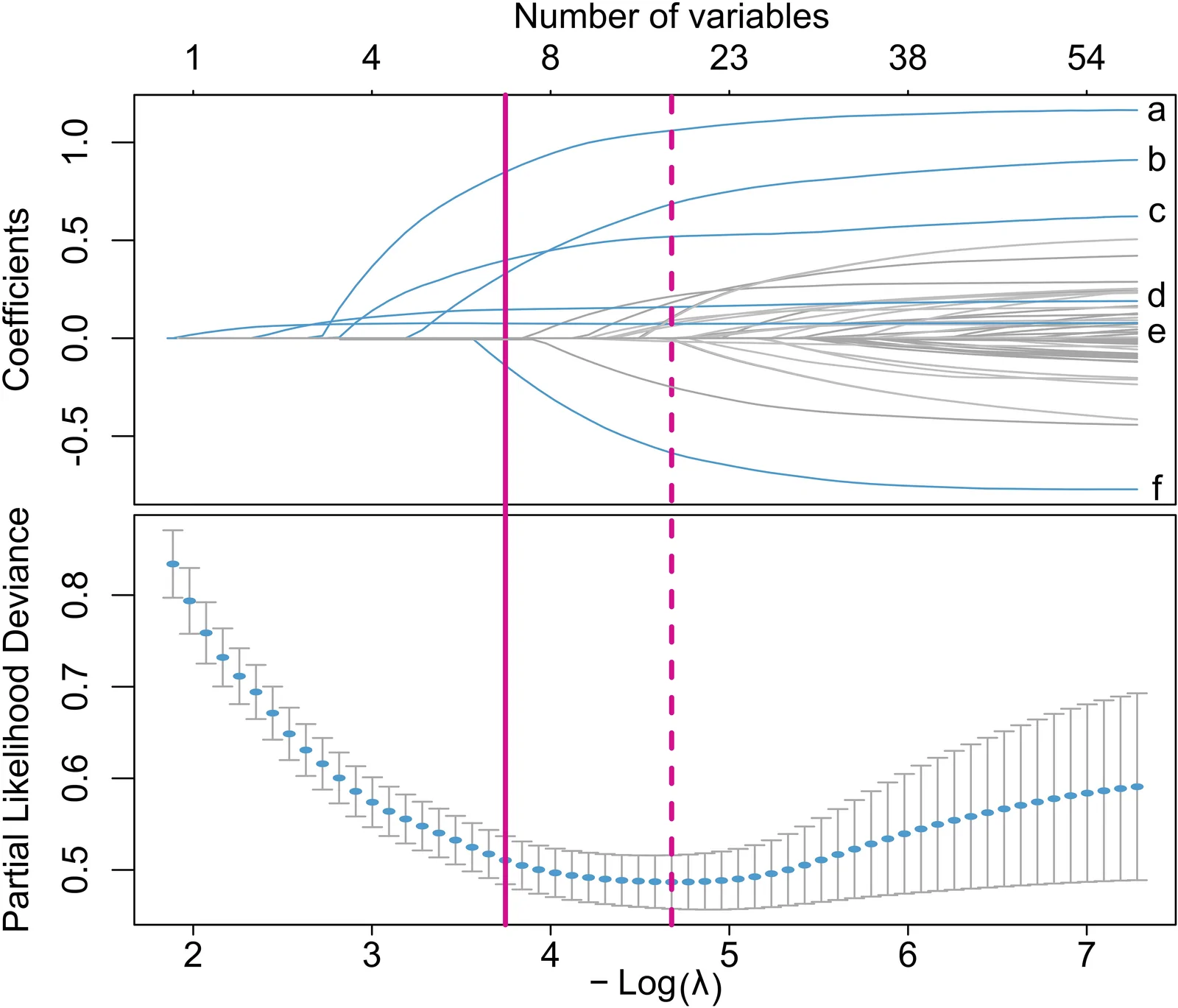
Screening process for potential predictors by LASSO method (The purple vertical dashed line shows the λ value at the minimum MSE, while the solid line shows the λ value at the minimum MSE + SE. The labels of blue curves from “a” to “f” represents the predictors of priority antibiotic combinations, SOFA, haematological malignancy, NLR, pre-ICU corticosteroid dose and halo sign, in turn).

**Table 2:** Predictor effects from the Cox and Fine-Gray models in the derivation cohort.

Predictors	Cox model		Fine-Gray model	
	HR (95%CI)	*P* value	sHR (95%CI)	*P* value
SOFA (non-linear) ^a^	–	< 0.01	–	< 0.01
Haematological malignancy	1.85 (1.45, 2.37)	< 0.01	1.82 (1.42, 2.33)	< 0.01
NLR (non-linear) ^a^	–	0.002	–	0.009
Halo sign	1.50 (1.18, 1.92)	0.002	1.51 (1.18, 1.93)	< 0.01
Priority antibiotic combinations	1.90 (1.54, 2.34)	< 0.01	1.90 (1.55, 2.32)	< 0.01
Pre-ICU corticosteroid cumulative dose (non-linear) ^a^	–	< 0.01	–	< 0.01

^a^ *P* value for overall non-linear association by Likelihood Ratio test.

**Figure 3:**
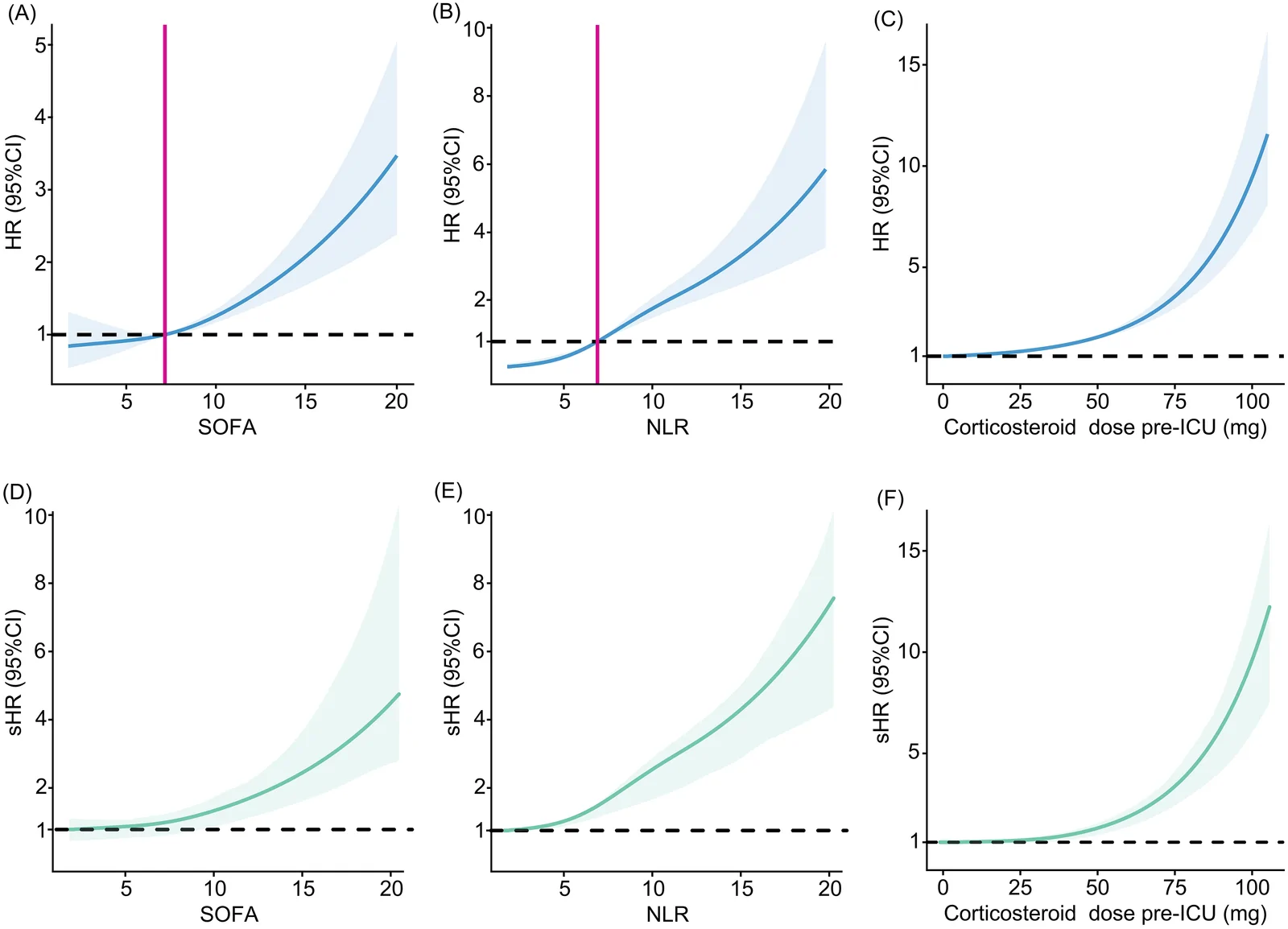
Non-linear associations of continuous predictors with VAPA risk in the derivation cohort (**(A–C)** show the HR for SOFA, NLR, and pre-ICU corticosteroid dose from the Cox model, respectively; **(D–F)** show the sHR for these predictors from the Fine-Gray model; the black dashed line indicates HR (sHR) = 1; shaded areas represent 95% CIs; red vertical lines indicate thresholds [HR = 1] per the Cox model).

### Models’ performance

The overall predictive performance in the derivation and validation cohorts is summarized in **Figure 4**. In the derivation cohort, discrimination did not differ significantly between the Cox and Fine-Gray models at either 7 days (DeLong’s D = -0.26, *P* = 0.798) or 14 days (D = -0.03, *P* = 0.974). Calibration plots indicated that the Fine-Gray model was better calibrated overall than the Cox model at both time points. Both models slightly underestimate VAPA risk (by 5%) in the low risk decile, whereas the Cox model overestimated VAPA risk in high risk decile and by 20–30% in the highest risk decile; the Fine-Gray model showed substantially less overestimation (by 1–2%) in the highest risk decile.

**Figure 4:**
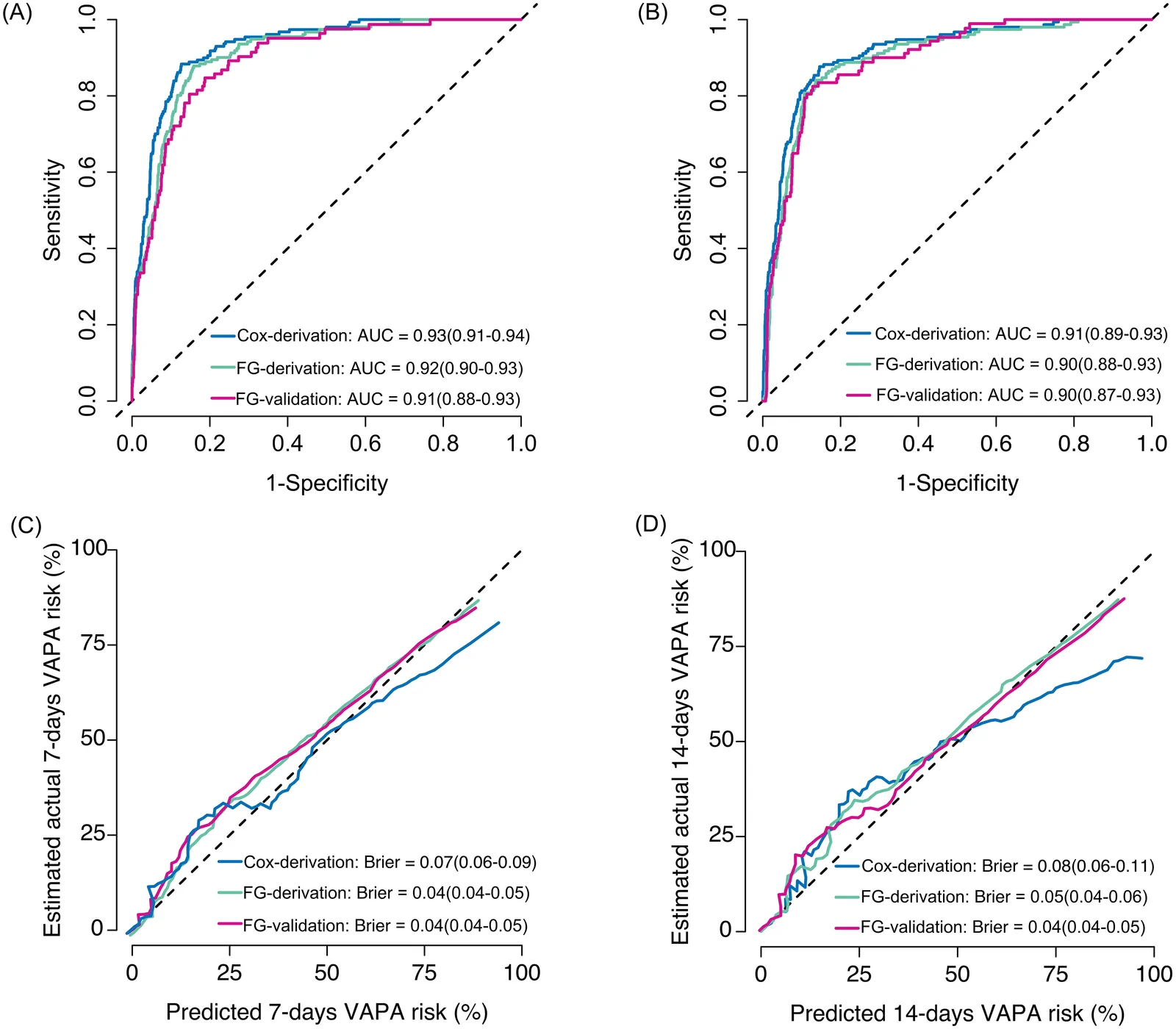
Overall performance for predicting VAPA in the derivation and validation cohorts [**(A, B)** show the time-dependent ROC curves at 7 and 14 days post-ICU admission, respectively; **(C, D)** show the calibration plots at 7 and 14 days post-ICU admission, respectively; the dashed line represents the ideal calibration line].

The Fine-Gray model demonstrated robust generalizability in the validation cohort. Its discriminatory performance remained high and showed no statistically significant decline compared to the derivation cohort at 7 days (D = 0.08, *P* = 0.937) or 14 days (D = 0.19, *P* = 0.853). The model maintained excellent calibration in the external cohort at both time points (Brier scores ≤ 0.05).

### Interaction and subgroup analysis

A significant interaction was observed between viral subtype and pre-ICU corticosteroid dose (*P* = 0.006), with a stronger association in COVID-19 patients (sHR per 10 mg increase in pre-ICU cumulative dexamethasone equivalent dose: 1.17, 95% CI: 1.09-1.26; **Figure 5**). No other significant interactions between viral subtype and the remaining predictors were observed (*P* > 0.05). Subgroup analysis showed consistent model performance across viral subtypes (**Figure 6**). Discrimination was similar between influenza and COVID-19 patients at both 7 days (D = 1.01, *P* = 0.313) and 14 days (D = 1.22, *P* = 0.224). Calibration curves indicated good agreement between predicted and observed probabilities in both subgroups at each time point (Brier scores ≤ 0.05).

**Figure 5:**
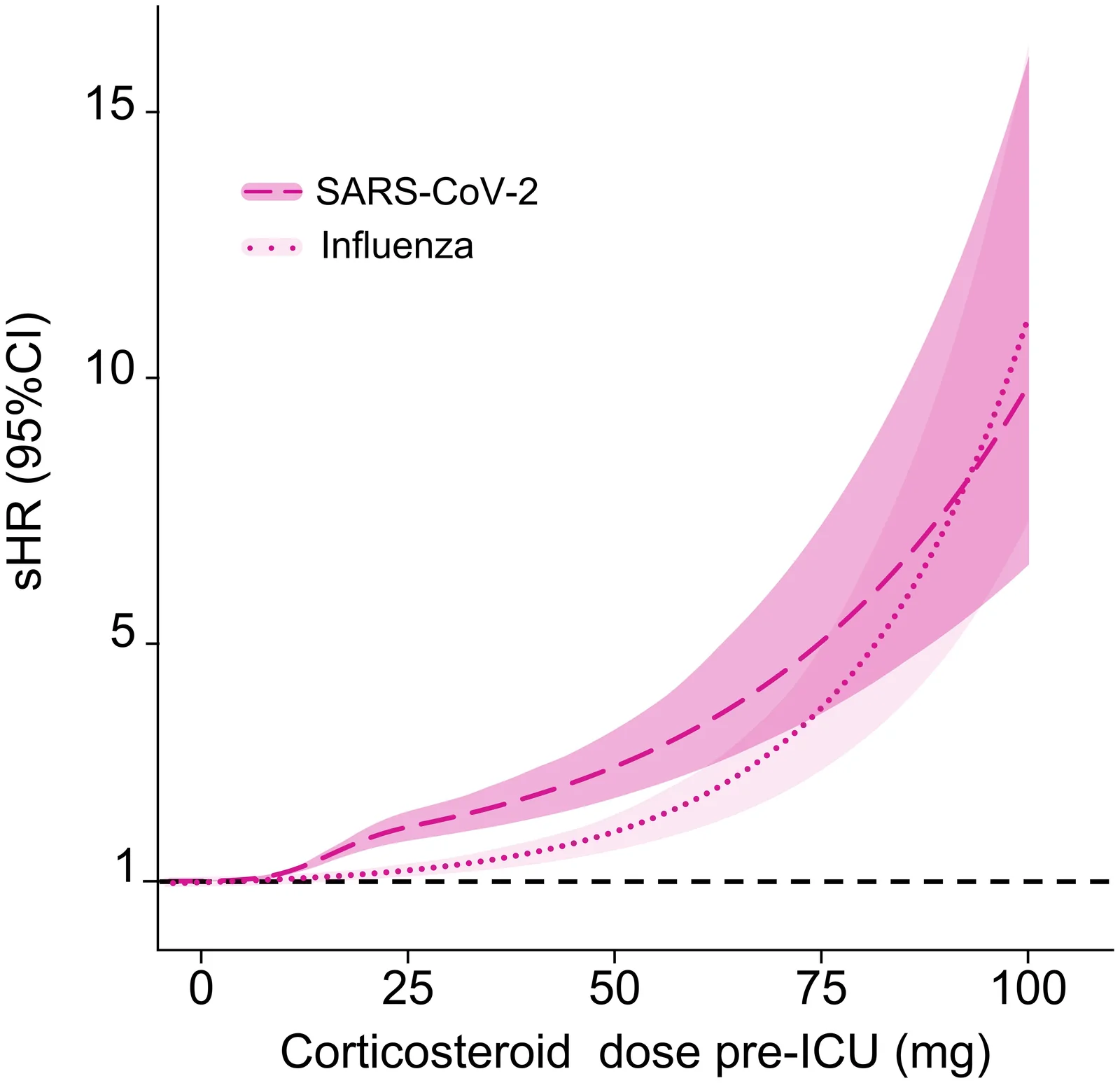
Virus-specific nonlinear association of pre-ICU corticosteriod dose with VAPA risk (the black dashed line represents sHR = 1; shaded areas represent 95% CIs).

**Figure 6:**
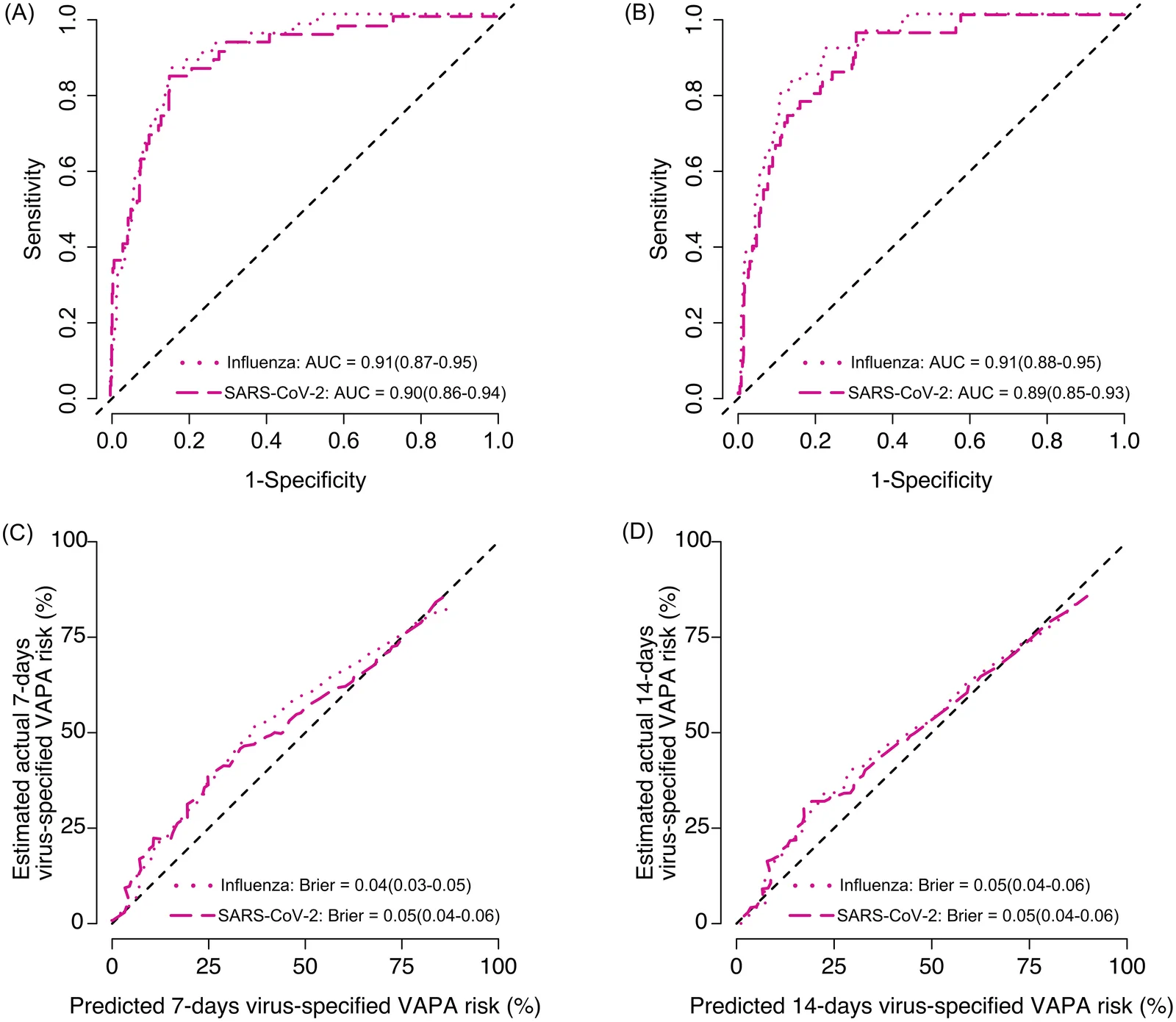
Virus-specific Fine-Gray models’ performance for predicting VAPA in the validation cohort [**(A, B)** show the time-dependent ROC curves at 7 and 14 days post-ICU admission, respectively **(C, D)** show the calibration plots at 7 and 14 days post-ICU admission, respectively].

Subsequently, an interactive, web-based dynamic prediction tool was deployed and is available via https://no57.shinyapps.io/VAPA_prediction/. The application features an intuitive interface with adjustable sliders for entering patient-specific variables, which then provides clinicians with a personalized VAPA risk probability and corresponding risk-stratified management recommendations in real time. Designed for real-time, point-of-care use without statistical expertise, the tool is alternatively accessible by scanning the quick response (QR) code provided in **Supplementary Figure 1**.

## Discussion

### Major findings and advantages

This study developed and validated a dynamic competing risk model for predicting the VAPA risk in critically ill patients with viral pneumonia through a multi-center, ambispective cohort. The model employed the Fine-Gray subdistribution hazards framework, accounting for non-VAPA death, effectively avoided the overestimation of VAPA risk observed in the standard Cox proportional hazards model. Our model demonstrated excellent discrimination and calibration in both the derivation and the prospective temporal validation cohort, with consistent performance across different viral subtypes (influenza and COVID-19). More importantly, we were the first to identify a significant J-shaped association between the pre-ICU cumulative corticosteroid dose and VAPA, observing a stronger effect in COVID-19 patients than in influenza patients. This may inform risk stratification and individualized consideration of immunomodulatory strategies in the context of different pathogens. This study not only addresses the methodological and generalizability gaps in existing prediction tools but also, by deploying the final model as a real-time clinical prediction tool, lays a solid foundation for achieving precise risk stratification and targeted intervention for VAPA.

### Comparison and explanations

In this multi-center cohort of critically ill patients, the overall incidence of VAPA was 8.8%. Within this composite outcome, the IAPA-specific incidence was consistent with previous reports (Shi et al., 2022; Lu et al., 2024). In contrast, the CAPA-specific incidence was substantially higher than the approximately 6.0% documented in studies from the pandemic period, while aligning with recent reports from the post-vaccination era, where incidence rates exceeding 10% have been observed (Prattes et al., 2022; Bay et al., 2024; Gioia et al., 2024). This temporal disparity likely reflects a fundamental shift in the at-risk population. Early CAPA epidemiology was primarily derived from studies involving predominantly immunocompetent patients admitted to the ICU (Gioia et al., 2024). Our cohort, however, was recruited from January 2023 to June 2025, a period initiated after the comprehensive lifting of COVID-19 containment policies in China. It thus represents the clinical landscape of the post-vaccination era, characterized by a critically ill COVID-19 population that frequently includes individuals with significant comorbidities that compromise the development of a protective immune response—even in vaccinated patients—thereby heightening susceptibility to aspergillosis (Feys et al., 2024).

The clinical implications of our findings for VAPA risk prediction in critically ill patients are significant but not straightforward. Overestimation of VAPA risk by the Cox model was most pronounced in the high-risk stratum, with an overestimation of 20–30% —precisely the patient group for whom clinical decisions are most critical, and where predicted risk often exceeds the threshold for initiating empirical antifungal therapy. In such high-stakes scenarios, substantial overestimation could lead clinicians to administer unnecessary antifungal treatment to patients not truly at high risk, resulting in drug-related toxicities, increased healthcare costs, and the potential for driving antifungal resistance. In contrast, the Fine-Gray model, which closely approximates the actual observed probability of VAPA, is unlikely to alter management for genuinely high-risk patients in most clinical situations. Consequently, the more accurate risk estimation afforded by the Fine-Gray model enables clearer risk communication between clinicians and patients. This improved accuracy not only helps refine the targeting of preemptive therapy but may also better inform patient preferences regarding preventive treatment, based on a more precise understanding of their personalized risk–benefit profile.

The extent of overestimation of the target event risk by the Cox model depends on the incidence of competing events (Cooper et al., 2022). Patients may experience competing events—such as death from other causes (e.g., underlying malignancy, acute cardiovascular events)—rather than the endpoint of VAPA. Cox model typically treats non-VAPA death as censored, implicitly assuming that these patients remain at risk for VAPA in the future. This leads to an overestimation of the actual VAPA risk because individuals who have died from competing events no longer have any opportunity to experience VAPA (Nolan and Chen, 2020). Notably, longer prediction windows for VAPA (e.g., extending from 7 to 14 days post-ICU admission) increase the opportunity for patients to die from other causes. Our results demonstrate that the degree of VAPA risk overestimation with the Cox model becomes more pronounced as the prediction horizon lengthens, underscoring the importance of using methods such as the Fine-Gray model to account for competing risks in this setting.

Notably, our analysis revealed a nuanced relationship between viral subtype and model stability. While a statistically significant interaction was identified between viral subtype and pre-ICU corticosteroid dose, this biological effect modification did not meaningfully affect overall model performance. Clinically, the consistent performance across viral subtypes supports the generalizability of our prediction tool for both influenza and COVID-19. Nevertheless, the observed interaction indicates that the relationship between pre-ICU corticosteroid exposure and VAPA risk is virus-specific, with a stronger association evident in COVID-19 patients than in those with influenza. This pathophysiological distinction, though not detrimental to model accuracy, offers valuable insight for tailoring therapeutic strategies. Our findings highlight the importance of comprehensive validation that incorporates both statistical interaction testing and clinical performance assessment. The dissociation between significant interaction terms and preserved model performance underscores that the presence of effect modification does not necessarily compromise predictive accuracy in multi-variable models. This has implications for predictive modeling in heterogeneous populations, suggesting that a model can remain clinically applicable even when underlying risk mechanisms differ across subgroups.

Moreover, we identified NLR as a key predictor of VAPA, a finding pathophysiologically plausible. Elevated NLR captures both hyperinflammation (reflected by neutrophilia) and immunosuppression (reflected by lymphopenia)—a critical state of immune dysregulation often described as an “immunological paradox.” This coexistence of a “cytokine storm” and impaired cellular immunity creates an ideal environment for invasive fungal infections (Santos et al., 2024). Furthermore, the positive association between corticosteroid use and invasive pulmonary aspergillosis is well established and was also confirmed in our study (Working group for the surveillance and control of COVID-19 in Spain, 2020; Hashim et al., 2022). And growing evidence suggests that corticosteroids may not benefit a substantial proportion of patients with severe influenza or COVID-19 (Short et al., 2014; de Almeida et al., 2022; Hoenigl et al., 2022). Given these insights, we recommend that clinicians individualize the decision to administer corticosteroids in patients with severe viral pneumonia and consider screening for aspergillosis in high-risk settings.

Another finding of clinical relevance pertains to the role of antifungal prophylaxis in VAPA, which remains controversial and is not substantiated by robust evidence (Feys et al., 2024). Consistently, our analysis further supports this view, as no significant protective effect of prophylaxis was observed. This lack of efficacy in IAPA is likely attributable to its frequent early onset following ICU admission, often rendering prophylaxis initiated too late to be effective (Van De Veerdonk et al., 2017). Our group recently confirmed, via a target trial emulation approach, that early antifungal prophylaxis does not reduce IAPA incidence and is associated with hepatotoxicity, further challenging the universal prophylaxis strategy (Li et al., 2026). In contrast, CAPA typically occurs later in the ICU stay, theoretically allowing a wider window for intervention. Although a recent qualitative systematic review noted encouraging results from several small retrospective studies, these are limited by considerable potential for bias and, crucially, have not consistently translated into a survival benefit (Hawes and Permpalung, 2022). Therefore, considering our findings—alongside the high cost, significant toxicity, and drug–drug interactions associated with first-line azoles such as voriconazole—we suggest that the widespread implementation of antifungal prophylaxis for VAPA is currently unjustified (Vanderbeke et al., 2021). Future strategies should instead prioritize risk stratification based on local epidemiology, validated biomarkers, and clinical predictors, as examined in our study, to identify patients most likely to benefit, rather than adopting a universal prophylactic approach.

### Limits

This study has several limitations. First, although the model underwent internal and external validation within a large multi-center cohort from Central China, its generalizability requires further verification in populations from different geographical regions, ethnicities, and healthcare systems; independent external validation using multi-national cohorts is therefore a priority for future research. Second, as our cohort primarily comprised patients with influenza and COVID-19, the predictive performance for pulmonary aspergillosis following pneumonia caused by other respiratory viruses, such as respiratory syncytial virus or adenovirus, remains unclear. Future work will aim to extend the model to these populations as relevant data become available. Finally, as a non-interventional investigation, this study validated the model’s predictive performance only; its clinical utility—specifically, its overall impact on patient benefit—needs to be further assessed through interventional studies, such as randomized controlled trials comparing model-guided antifungal initiation versus routine care, which represents a key direction for our future research.

## Conclusions

In this multi-center, ambispective cohort study, we developed and validated a novel, dynamic competing-risk prediction model for VAPA in critically ill patients with influenza or COVID-19. This model, based on the Fine-Gray subdistribution hazards framework, effectively addresses the overestimation of risk inherent in standard time-to-event analyses by accounting for the competing risk of non-VAPA death, and further captures non-linear relationships between key continuous predictors and VAPA risk through the use of RCS. The Fine-Gray model showed discrimination similar to the Cox model but improved calibration, with consistent performance across viral subtypes. Accordingly, we implemented a practical tool to facilitate personalized, preemptive management of VAPA.

## Data Availability

The raw data supporting the conclusions of this article will be made available by the authors, without undue reservation.
